# Recent Progress in Metal Oxide-Based Photocatalysts for CO_2_ Reduction to Solar Fuels: A Review

**DOI:** 10.3390/molecules28041653

**Published:** 2023-02-09

**Authors:** Xuanzhen Li, Jing Xiong, Zhiling Tang, Wenjie He, Yingli Wang, Xiong Wang, Zhen Zhao, Yuechang Wei

**Affiliations:** 1 State Key Laboratory of Heavy Oil Processing, College of Science, China University of Petroleum, Beijing 102249, China; 2 Key Laboratory of Optical Detection Technology for Oil and Gas, China University of Petroleum, Beijing 102249, China

**Keywords:** photochemistry, CO_2_ reduction, metal oxide materials, solar fuels, macroporous structure

## Abstract

One of the challenges in developing practical CO_2_ photoconversion catalysts is the design of materials with a low cost, high activity and good stability. In this paper, excellent photocatalysts based on TiO_2_, WO_3_, ZnO, Cu_2_O and CeO_2_ metal oxide materials, which are cost-effective, long-lasting, and easy to fabricate, are evaluated. The characteristics of the nanohybrid catalysts depend greatly on their architecture and design. Thus, we focus on outstanding materials that offer effective and practical solutions. Strategies to improve CO_2_ conversion efficiency are summarized, including heterojunction, ion doping, defects, sensitization and morphology control, which can inspire the future improvement in photochemistry. The capacity of CO_2_ adsorption is also pivotal, which varies with the morphological and electronic structures. Forms of 0D, 1D, 2D and 3DOM (zero/one/two-dimensional- and three-dimensional-ordered macroporous, respectively) are involved. Particularly, the several advantages of the 3DOM material make it an excellent candidate material for CO_2_ conversion. Hence, we explain its preparation method. Based on the discussion, new insights and prospects for designing high-efficient metallic oxide photocatalysts to reduce CO_2_ emissions are presented.

## 1. Introduction

According to the report from Carbon Emission Accounts and Datasets for emerging economies (CEADs), China has emitted more than 10 billion tons of CO_2_ per year since 2018. It is inextricably linked to people’s desire for a more comfortable and convenient life. Nearly 60% of total emissions emanated from the production of cement, steel and coal-fired power generation processes. As a result of the greenhouse effect caused by ever-growing CO_2_, the global average temperature is continuously increasing [1] and various types of severe weather will incur more frequently, such as ice melting, land drought, typhoons, hurricanes, earthquakes and tsunamis [2]. It is, therefore, imperative to convert and utilize the CO_2_ present in the atmosphere.

The transformation of CO_2_ can be driven by illumination, electricity and heat [3]. Solar energy is a safe, clean, renewable and inexhaustible energy source, so it is ingenious to achieve this conversion with sunlight [4]. Additionally, the reaction conditions in photocatalytic processes are mild [5]; thus, it is easy to conduct experimental tests. A new world opened up since H_2_ and O_2_ were obtained after radiating TiO_2_ with light. Extensive research about photocatalyst were conducted until the year of 2000. Since then, a growing number of materials have been designed and studied to absorb solar energy, including oxide semiconductors, sulfides (ZnS, CdS and MoS_2_), and polyoxometalates (Bi_2_WO_6_, Bi_2_MoO_6_ and BiFeO_3_) [6,7]. Organics, organometallic complexes, covalent organic polymers and noncovalent self-assembled supramolecular organic matter are also involved. Among them, inorganic metal oxide materials are widely studied for establishing efficient artificial photosystems due to their low cost, facile synthesis, stable crystal structures and environmental friendliness. TiO_2_, Cu_2_O, ZnO, WO_3_ and CeO_2_ show promising research value as the most common materials.

Despite the significant progress that has been achieved, researchers still have difficulties in developing highly active catalysts because of poor light-harvesting capacity, low CO_2_ adsorption capacity and rapid recombination of charge carriers. To this end, theoretical foundation and strategies to upgrade photocatalysts are described in detail in this article. Firstly, the advantages of exposed facets adjustment, and the morphology of 0D, 1D, 2D and 3DOM materials are summarized. Then, common co-catalyst materials are introduced. Defects, ion doping and sensitization engineering are also discussed. We provide a basic understanding on these approaches to inspire the future improvement in photocatalytic field. Furthermore, as inorganic metal oxide materials are more suitable for large-scale production, this paper can serve as a model for future industrialization.

## 2. Theoretical Foundation and Strategies of Photocatalytic CO_2_ Reduction

**Figure 1:**
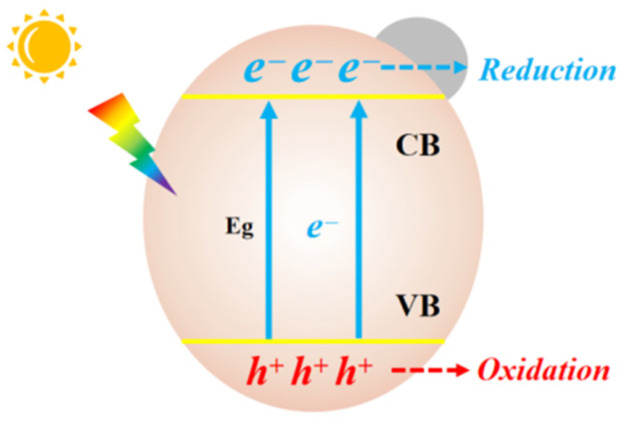
Fundamentals of photocatalytic reduction on a semiconductor catalyst.

The photocatalytic process in semiconductors can generally be described as shown in Figure 1. Upon being excited by an incident photon with energy equal to or higher than the bandgap (*E*g), charge carriers are generated. Electrons (e^−^) at the bottom of conduction band (CB) migrate to the surface of the catalyst to initiate reduction reactions with CO_2_. Holes (h^+^) at the top of valence band (VB) conduct oxidative reactions.

The photocatalytic process of CO_2_ conversion on semiconductors can be divided into five general steps: (1) Formation of electron–hole pairs under light radiation, (2) Separation and migration of the electrons and positive holes, (3) Adsorption and activation of CO_2_, (4) Redox reactions that between surface-adsorbed species and electron–hole pairs, and (5) Desorption of the product. The electrochemical reactions with standard oxidation–reduction potentials (at pH 7 vs. NHE) are as follows:

H_2_O + h^+^ → OH• + H^+^ (1)

H^+^ + e^−^ → H• (2)

H• + H• → H_2_ (3)

CO_2_ + e**^−^** → CO_2_**^−^** (4)

CO_2_ + 2H^+^ + 2e^−^ → HCOOH, E_0_ = −0.61 V (5)

CO_2_ + 2H^+^ + 2e^−^ → CO + H_2_O, E_0_ = −0.53 V (6)

CO_2_ + 4H^+^ + 4e^−^ → HCHO + H_2_O, E_0_ = −0.48 V (7)

CO_2_ + 6H^+^ + 6e^−^ → CH_3_OH + H_2_O, E_0_ = −0.38 V (8)

CO_2_ + 8H^+^ + 8e^−^ → CH_4_ + 2H_2_O, E_0_ = −0.24 V (9)

**Figure 2:**
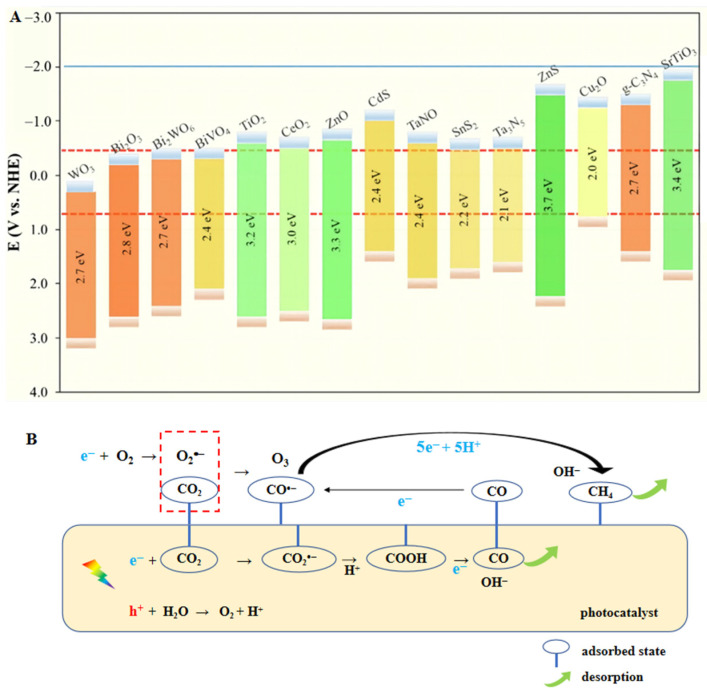
(**A**) Band edge positions of various semiconductors in relation to the redox potential of different products at pH = 7 [11]. (**B**) Schematic illustration of the mechanism for CH_4_/CO generation.

To carry out the conversion, it is necessary to comprehend the structure of CO_2_. Due to the great symmetry and high bond energy of 750 kJ/mol, it is particularly difficult to break the C=O double bond. Therefore, the transmutation between CO_2_ and bent radical anion of CO_2_^•−^ on the surface of the catalyst is widely recognized as the first step to activate CO_2_ for subsequent reaction. Additionally, photoreactions occur favorably only when the CB position of the catalyst presents a more negative potential than the target reduction and the VB position is more positive than the oxidation reaction. Figure 2A displays the CO_2_ reduction potentials of some common semiconductors, along with the *E*g positions. From a molecular perspective, the adsorbed CO_2_ is combined with e^−^, H^+^ or other intermediates on the surface of the catalyst. On the basis of newly-released reports, the photocatalytic mechanism of CO_2_ reduction to CH_4_/CO is graphically explained in Figure 2B [8,9,10].

Therefore, enough e^−^ and H^+^ are requisites for the entire photoreduction process. A series of fuels can be obtained by means of well-designed catalysts, such as CH_4_ [12,13], CH_3_OH [14] and HCOOH [15]. It makes sense to create valuable solar fuel from CO_2_, which provides a solution to environmental pollution. Products, alcohols, hydrocarbons and even carbon monoxide can be used as feedstock for energy reserves or high-value compounds.

**Figure 3:**
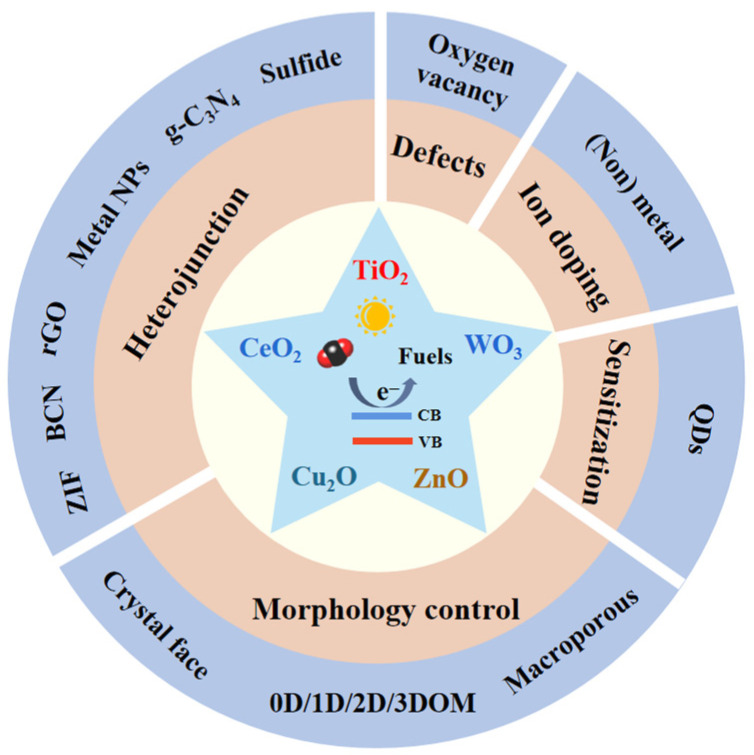
Strategies to boost the photocatalytic CO_2_ reduction.

Figure 3 summarizes the current strategies that can boost the photocatalytic CO_2_ reduction pace, with detailed information being presented sequentially below.

### 2.1. Morphology Control

The surface topography of nanocrystals could evidently alter the electronic structure, surface energy and chemical properties of catalysts. Therefore, morphology control is one of the most important issues that concerns researchers in nanoscience, chemistry and physics. Open facets and edges determine the shape of nanocrystals. Thus, the preferential adsorption of additives on certain crystal surfaces provides a good opportunity to tune the surface of nanomaterials.

#### 2.1.1. Exposed Facet Adjustment

Exploring and figuring out the variation that is connected with exposed surfaces is crucial to elucidate shape-related chemical and physical properties. In semiconductor crystals, different facets have distinct electronic band structures that influence the transport of photoexcited carrier charges. It is wise to expose active facets to tune the CO_2_ photoreduction efficiency [16].

#### 2.1.2. Quantum Dots (QDs)

Zero-dimensional (0D) semiconductor quantum dots (QDs) have many unique properties, such as quantum confinement effect, high extinction coefficient and multiple exciton generation [17]. Hence, QDs show much better photoactivity in the visible light region. Unlike bulk materials, surface atoms make up the majority of QD semiconductors. The abundant surface sites enhance the interaction between electron donors and acceptors, thus facilitating the photocatalytic charge transfer rate.

#### 2.1.3. One-Dimensional and Two-Dimensional Structures

One-dimensional nanostructured catalysts have high aspect ratios, such as nanowires, nanorods and nanotubes. The morphological tuning of the material makes a difference to their thermal, optical, electrical and magnetic properties [18]. For instance, the TiO_2_ nanotube can act as a channel for electron transfer and build up the chemical reactions rate.

Two-dimensional layered materials can protect a tiny particle component from aggregating. The CO_2_ adsorption capacity on the surface of 2D photocatalysts can be enhanced due to the large specific surface area and bountiful surface defects [19]. Two-dimensional lamellar nanosheets are widely used in photocatalysts, such as g-C_3_N_4_, MoS_2_ and WO_3_.

#### 2.1.4. Macroporous and Three-Dimensional Ordered Macroporous (3DOM) Structures

Macroporous materials are being widely used in massive photocatalytic materials, owing to their excellent properties [20]. Unlike dispersed particles, sunlight can penetrate the pore wall easily and scatter widely inside the hollow structure, thus increasing the efficiency of illumination. Subsequently, the slender walls of pore reduce the transfer length of photo-generated charge carriers. Electrons (e^−^) and holes (h^+^) are separated more efficiently when heterojunctions are loaded on porous materials. The specific surface it provides is so large that more CO_2_ molecules have a chance to contact the catalyst for reduction reactions.

Growing attention has been paid to hierarchical composite pores, including photonic crystal catalysis and separation of sub-microns. The slow light effect of photons associated with 3DOM materials have been considered to increase solar radiation absorption and enhance photocatalyst performance [21]. 3DOM products with periodic macrostructures [22], known as inverse opal, have been applied in battery materials, sensors, separation engineering and heterogeneous catalysis. Thanks to the periodic dielectric constants, Bragg diffraction permits certain wavelengths of light to radiate, leading to stop-band reflection. The limited photons reflected back will slow down at the edge of the stop bands, from which it received its name of “slow photons” [23,24]. It accelerates the light absorption rate when the photon energy is consistent with the absorption spectrum of the 3DOM materials [25]. In addition, the light scattering properties are optimized due to the relation between the macrospores and pore walls. For example, a 3D macro mesoporous Mo: BiVO_4_ architecture [26] was designed and fabricated, which had a superior photocurrent density.

It has been demonstrated that gas absorption and separation functions benefit from orderly porous architectures [27]. 3DOM chemicals show superior CO_2_ absorption/desorption rates than other commercial products, as the special structure can capture CO_2_ from the ambient air by utilizing humidity variation. It is a great way to use microporous catalysts because it not only increases visible light absorption but also shortens the transport time of CO_2_. They are available at a low cost through simple and rapid methods.

#### 2.1.5. Preparation of 3DOM Materials

**Figure 4:**
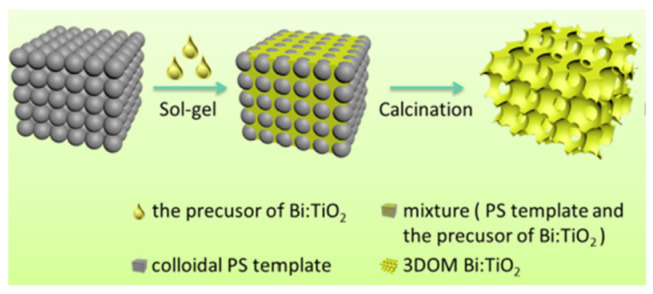
The fabrication process of the 3DOM Bi-doped TiO_2_ [26].

Specifically, 3DOM materials are produced by the colloidal crystal template (CCT) method (Figure 4). A uniform and close-connected organic sphere template can be obtained, firstly, through three key processes. Then, seep metallic salt sol into the void of microspheres. After heat treatment, the organic microsphere template fades away and leaves a metal oxide frame. (i) The polymerizable monomer (methyl methacrylate, styrene) and initiator are mixed and heated under the protection of Ar; (ii) the earlier reaction liquid is filtered with microfiltration membrane; (iii) the microsphere mixture is centrifuged at a high speed for a long time, yielding the polymethylmethacrylate (PMMA) or polystyrene (PS) template; (iv) the template is immersed in the precursor solution and (v) is calcinated.

In the absence of ultra-high temperature and expensive equipment, a fantastic 3DOM structure was obtained. Various materials of frame can also be synthesized by altering the precursor formula.

### 2.2. Heterojunction

Photogenerated charge carriers in a single material tend to recombine rapidly due to coulombic force and they can be separated efficiently in the multi-materials that are in close contact [28]. The heterojunction is an interface between various semiconductors with different energy band structures. The heterojunction structure can reduce the probability of charge recombination and is therefore considered an effective method to enhance the photocatalytic activity [29,30].

### 2.3. Defects

All semiconductors have surface defects, which are rooted in the absence of host atoms. The amount of oxygen vacancies can be altered by ion doping and nanocrystal modifications. Oxygen defects and other defects originating from the generated vacancy [31] can facilitate the separation of h^+^/e^−^. Over and above, defects can also activate CO_2_ molecules, reducing the activation energy of the reaction [32].

### 2.4. Ion Doping

Elemental doping is a common strategy to modulate the surface electronic structure. Then, the band gap of the semiconductors make a difference, nonmetal doping (such as N, C and O) mainly alters the VB and metal-doping (e.g., Mo, Co and Ni) influences the CB [33,34]. For example, Cu-doped TiO_2_ absorbs more visible light due to the Cu 3d-Ti 3d optical transition [35].

### 2.5. Sensitization

Sensitization means coupling the quantum dots, dyes, etc., with semiconductors to increase the photogenerated carriers and promote the absorption of light by taking advantage of their receptivity in UV, visible or infrared light.

In the following, exuberant measures to optimize TiO_2_-, WO_3_-, ZnO-, Cu_2_O- and CeO_2_-based photocatalysts for CO_2_ reduction are demonstrated.

## 3. Photocatalysts with Different Basis Matrices

### 3.1. TiO_2_-Based Photocatalysts

Titanium dioxide (TiO_2_) is notable in photochemistry, with advantages such as non-toxic, cheapness, corrosion resistant, good physical and chemical stability. However, owing to the wide *E*g of 3.0–3.2 eV, TiO_2_ only absorbs energy in the ultraviolet region (3–5% of the solar energy), and photoexcited charge pairs are easy to combine, resulting in quantum inefficiency. Usually, TiO_2_ is divided into the rutile phase and anatase phase on the basis of atomic arrangement modes. Rutile TiO_2_ is thermodynamically stable and does not distort or decompose at high temperatures. It has a narrower energy gap (3.0 eV) and a wider spectral response than the anatase phase (3.2 eV). Rutile TiO_2_ seeds generally grow larger in size and tend to form an agglomerated structure. Smaller anatase TiO_2_ particles have a wider lattice gap and abundant surface oxygen defects, which make it favorable for ion doping and photoreactions.

#### 3.1.1. Morphology Control

**Figure 5:**
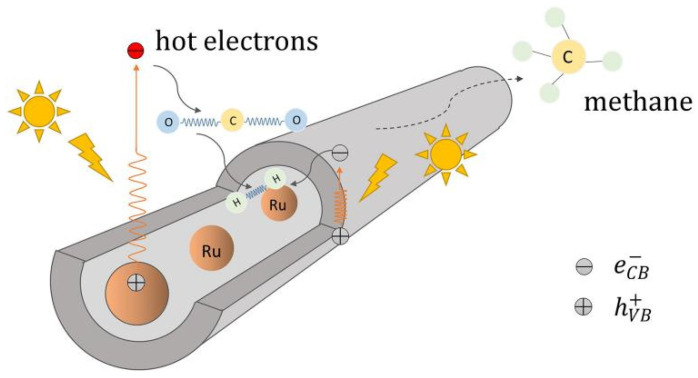
The reaction pathway for the Ru entrapped in the TiO_2_ nanotubes catalyzes CO_2_ methanation [37].

In hollow nanotube-shaped catalysts, the transport speed of CO_2_ and photoproducts can be facilitated. Ru phase is inclined to form methane in CO_2_ hydrogenation process [36] and Yang et al. [37] entrapped Ru nanoparticles in TiO_2_ nanotubes. Restricted Ru nanoparticles were resistant to sintering and leaching in the Ru-in/TNT catalyst channel (Figure 5). Electrons tend to gather in the tubes because of a confinement effect, which leads to an abundant, accessible metallic phase. It is easier for high-priced Ru species to combine with free electrons and then exhibit a superb CH_4_ and CH_3_OH yield.

On the contrary, Kar et al. [38] loaded metal nanoparticles onto vertically oriented 1D TiO_2_ nanotube arrays (TNAs) platforms via the graft method. In the synthesis process, Au, Ru and ZnPd NPs grow anodically on transparent glass substrates. There is no band bending phenomenon in Au NP-grafted TiO_2_, which can be observed from ultraviolet photoelectron spectroscopy (UPS). TPD experiments proved that all NP-grafted samples absorb more CO_2_ than TNAs. It is worth noting that, in nanoparticle-grafted TNAs, blue photons close to and below the TiO_2_ band edge were excited to drive CO_2_ photoreduction process. Ru-TNAs, ZnPd-TNAs and Au-TNAs had the CH_4_ formation rates of 26, 27 and 58 µmol·g^–1^·h^–1^, respectively. Pt and CoO_x_ growing on the outer and inner layers of a porous TiO_2_-SiO_2_ frame, respectively, can act as “warehouses” for e^−^ and h^+^, and the selectivity of the new system for reducing CO_2_ to CH_4_ can reach 94% [39].

Metal–organic frameworks (MOFs), also known as porous coordination polymers, composed of organic linkers and metal nodes (metal ions or clusters), are a kind of porous crystalline inorganic–organic hybrid materials. The unique advantages of MOFs, such as extremely high surface area, uniform adjustable porous structure and high density coordination unsaturated metal sites, have been extensively researched. They have been applied in many fields, such as gas adsorption and separation, sensing, catalysis, capture and conversion of CO_2_. The high specific surface area and uniform porous structure of MOFs make it possible to incorporate metal nanoparticles (as electron acceptors) into their frameworks, leading to efficient charge separation. In addition, the eminent CO_2_ adsorption capacity of various MOFs resulted in higher concentration of CO_2_ in the pores, which helped to accelerate the photoreaction. In [40], a porous material-zirconium-based organic skeleton (UiO-66) was introduced to a TiO_2_ photocatalyst as an effective CO_2_ adsorbent. The designed two-step strategy endowed the TiO_2_/UiO-66 composite with abundant graded pore structure, thus ensuring sufficient catalytic sites and high CO_2_ adsorption capacity (78.9 cm^3^ g^−1^). The ultrafine TiO_2_ nanoparticles were loosely loaded on the UiO-66 surface rather than tightly packed due to the electrostatic repulsion, thus ensuring the exist of microporous of the MOF. Finally, in the weak gas–solid catalytic system with water as the electron donor, the yield of CH_4_ was up to 17.9 μmol g^−1^ h^−1^, and the selectivity was 90.4%. Additionally, the photocatalytic efficiency was comparable to that of pure CO_2_ atmosphere even under low CO_2_ concentration conditions (≤2%).

##### Exposed Facet Adjustment

**Figure 6:**
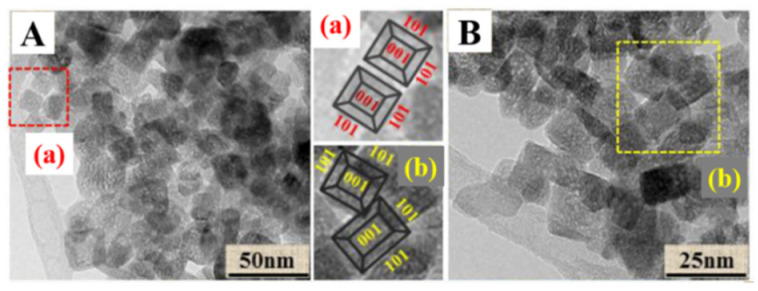
(**A**,**B**) HRTEM images of s-TiO_2_ (**a**,**b**) partial enlarged drawing [41].

A Pt-TiO_2_ single atomic site catalyst (PtSA/Def-s-TiO_2_) was prepared [41] by the “thermal solvent-argon treatment and hydrogen reduction” method. In order to construct Ti–Pt–Ti structures, TiO_2_ nanosheets with oxygen deficient sites were used to anchor monatomic Pt particles, which can retain the stability of isolated single atomic Pt and improve photocatalytic performance. The exposed (101) and (001) crystalline of TiO_2_ nanosheets (Figure 6A,B) were determined by transmission electron microscopy, and a thickness of 6.9 nm was observed through atomic force microscope. The EPR spectra of the samples confirm that the rich oxygen defect structure can be obtained by heating TiO_2_ nanosheets in argon atmosphere. They also indicated that single-atom Pt junctions were formed by occupying the oxygen defect sites, which provides a benchmark for the rational design of highly active and stable single-atom catalysts on metal oxide carriers with defect structures. CH_4_ product can be detected when copper oxide nanoparticles are mixed with mesoporous TiO_2_ nanorods in close contact [42]. Both the special crystal plane and porous structure contribute to furthering the CH_4_ yield.

##### 3DOM Structure Ti-Based Materials

A ternary 3DOM Bi-doped TiO_2_ photocatalyst decorated with carbon dots (CDs) was obtained, whose pore engineering of the 3DOM skeleton greatly promoted the response in the whole solar spectrum range [26]. It exhibits enhanced photocatalytic performance because of its excellent exquisite structure and high charge transfer efficiency. Similarly, a BiVO_4_/3DOM TiO_2_ nanocomposite [43] was synthesized as a highly efficient photocatalytic catalyst for the degradation of dye pollutants. Further studies on its textural, optical and surface properties revealed that connecting pores not only improve the electron transfer rate between coupled materials, but also provide abundant active sites for reactant molecules.

**Figure 7:**
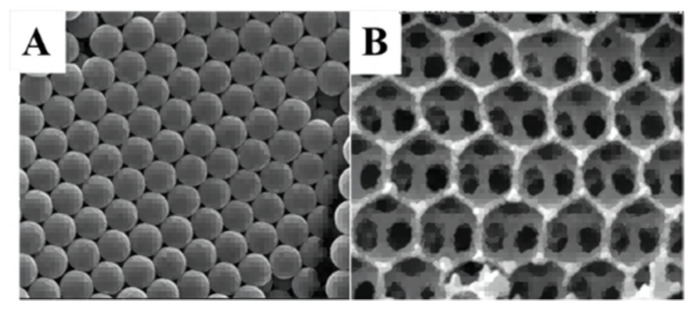
(**A**,**B**) SEM images of colloidal crystal template and CeO_2_/3DOM TiO_2_ [44].

3DOM TiO_2_ were prepared [44] by the CCT method (Figure 7A) and CeO_2_/3DOM TiO_2_ samples (Figure 7B) were obtained by the original bubble-assisted membrane precipitation method. The introduction of CeO_2_ nanolayers broadened the photo-absorption range and facilitated the separation of photogenerated electron−hole pairs. The mesoporous structure provided a larger surface area and the catalyst exhibited a higher CO_2_ reduction activity.

**Figure 8:**
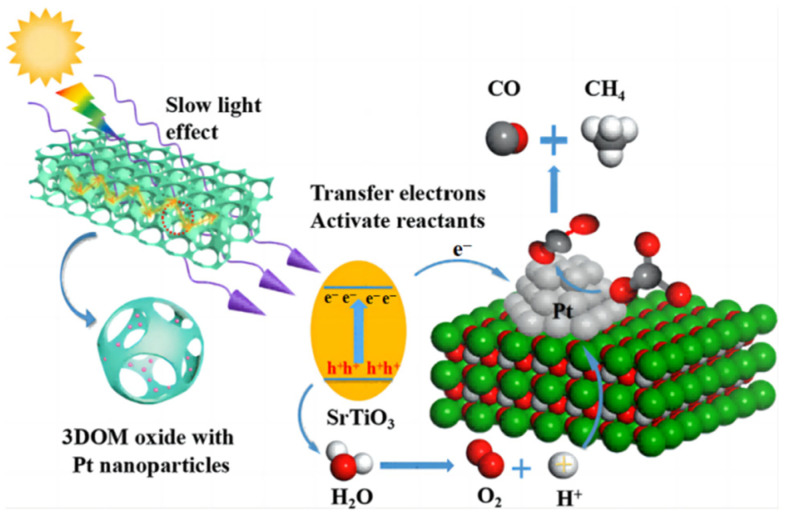
The possible mechanisms of the photocatalytic CO_2_ reduction over Pt/3DOM SrTiO_3_ [46].

Pt-particle-decorated 3DOM carbon-coated TiO_2_ and g-C_3_N_4_ were combined [45] to construct an all-solid-state catalyst for CO_2_ artificial photoconversion. Slow photon effect and carbon coat optimized the absorption capability of light. The exquisite design drove vectorial electrons from TiO_2_@C to Pt particles and then fell to g-C_3_N_4_, which facilitated the carrier separation. The Z-scheme that consist of two isolated systems has three components and converts CO_2_ to CH_4_ with H_2_O at a yield of 65.6 μmol g^−1^ h^−1^. To enhance the surface enrichment of CO_2_, Wu et al. [46] fabricated 3DOM perovskite-type Pt_n_/SrTiO_3_, in which Pt nanoparticles can take in photoelectrons from SrTiO_3_ and transfer CO_2_ to CO and CH_4_ (Figure 8). Pt_2_/3DOM SrTiO_3_ exhibited the highest CH_4_ yield of 26.7 μmol g^−1^ h^−1^.

A two-dimensional MoS_2_ layers/3DOM TiO_2_ photocatalyst was prepared [47] to form heterojunctions, which had a higher performance in the range of 420–900 nm. Cu single atoms were uniformly distributed in 3DOM TiO_2_ via the in situ method [48], which provides active sites for CO_2_ photoconversion. The main products tested in the gas–solid two-phase system were CH_4_ and C_2_H_4_ with the corresponding generation rates of 43.15 and 6.99 μmol g^−1^ h^−1^. Chen’s work provided a new perspective for improving the catalytic efficiency by regulating reaction conditions. Jiao et al. [49] loaded core-shell structure AuPd NPs in 3DOM TiO_2_ via one pot of the gas bubbling-assisted membrane reduction method to form a functional photocatalyst. The low Fermi level of AuPd NPs empowered the catalyst system to trap electrons and enhance the separation of charge pairs. Carbon-quantum-dot-decorated 3DOM CaTiO_3_ photocatalysts were duly obtained [50], exhibiting an apparent quantum efficiency (QAY). The macro–meso–microporosity structure provided improved charge carrier separation and transport, and it was explored in depth through density functional theory calculations (DFT) and finite difference time-domain simulations.

#### 3.1.2. Heterojunction

p–n heterojunction: A p–n heterojunction is formed by combining p-type and n-type semiconductors. Even without light irradiation, electrons can diffuse from an n-type semiconductor to a nearby p-type one, in the case of the combination of two materials. Correspondingly, the holes on the surface of a p-type semiconductor are transferred to the n-type one, which results in an efficient separation of charge carriers. A ZnFe_2_O_4_-modified TiO_2_ was synthesized by the hydrothermal method [51], and the p-n heterojunction system could reduce CO_2_ to methanol at a yield of 75.34 μmol g^−1^ h^−1^.

**Figure 9:**
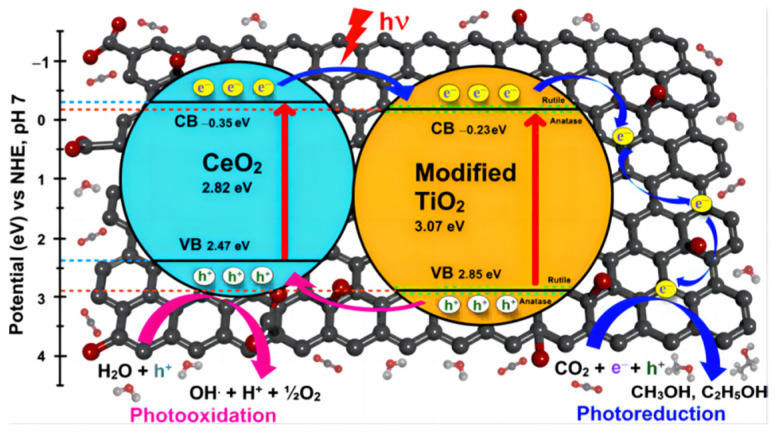
Mechanism for the photocatalytic conversion of CO_2_ with H_2_O to methanol and ethanol over TiO_2_/rGO/CeO_2_ photocatalysts [53].

rGO composite: In recent years, graphene materials have been widely used because of their large specific surface area, unique thermal stability and excellent electrical conductivity. Graphite nanomaterials are visible-light-responsive materials with appropriate band gaps, and the energy levels of CB and VB are in optimal positions relative to ordinary hydrogen electrodes. These unique photocatalytic properties have made them prime candidates for photocatalytic CO_2_ reduction. Fortunately, tightly contacted ultra-thin graphene layers and TiO_2_ compounds and can be prepared with some additives [52]. Seeharaj et al. [53] employed high-intensity ultrasonic waves (ultrasonic horn, 20 kHz, 150 W/cm^2^) to exfoliate the TiO_2_ surface, which led to a highly specific active area and highly reactive nanosheets. The modification of tiny rGO and CeO_2_ on the rGO nanosheet surface can improve the CO_2_ absorptivity and the charge carriers’ migration efficiency of the catalyst (Figure 9). A kind of d–π electron orbital overlap was formed between TiO_2_′s d orbitals and rGO’s π orbitals, which provides a good environment for activated CO_2_ and electrons. The complex heterojunction photocatalysts TiO_2_/rGO/CeO_2_ exhibited high yields of CH_3_OH (641 μmol/gcath) and C_2_H_5_OH (271 μmol/gcath).

BCN composite: The boron carbon nitride (BCN) composite BCN is an adjustable band gap material that has been applied in CO_2_ reduction, water splitting and as a detoxification catalyst. Highly negative CB potential of BCN materials makes them suitable for the construction of S-scheme heterojunction, and Kumar et al. [54] designed a novel S-scheme Fe@TiO_2_/BCN composite. In situ XPS technology, DFT calculations and finite difference time-domain simulations were adopted to verify the S-scheme transfer mechanism. Under visible light irradiation, the intimately contacted heterojunction reduced the probability of charge recombination. The sample exhibited excellent photocatalytic activity; in addition to converting CO_2_ selectively, it also degraded tetracycline antibiotics.

TMS composite: Some transition metal sulfides (TMSs) exhibit the same properties as Pt or Pd in photocatalyst. They form longer-lived charged carriers within the S_3p_ orbital [55] with a highly negative reduction potential. However, the self-oxidation of metal sulfides confuses researchers and limits their application. It can be alleviated by constructing a Z-scheme, and thus researchers introduced it to Bi-modified TiO_2_ [56]. Meanwhile, a spatially coupled heterojunction was enhanced by regularly capsulated CuCo_2_S_4_ yolk–shell hollow sphere.

#### 3.1.3. Ion Doping

In recent years, elements such as B, N, Co and Bi have been widely applied in TiO_2_ doping. A carbon-based hybrid nanocomposite reduced graphene oxide (rGO), belonging to the narrow band gap, with oxygen-containing functional groups on the surface that can be enhanced by π interactions [57]. Laminar graphene carriers not only prevent TiO_2_ repolymerization, but also hybridize the function of the catalytic system. Co-doped TiO_2_ was loaded on the rGO [58], and the Co peak in EDX spectra and C-O peak in FT-IR spectra confirmed the successful doping and the presence of graphene support, respectively. The size of TiO_2_ particles decreased from 48–80 nm to 23–28 nm, which is consistent with earlier reports of changes in titanium doping with transition metal ions.

#### 3.1.4. Sensitization

A growing number of semiconductor materials are being used to modify TiO_2_ dioxide, but randomly mixed catalysts are not stable enough to achieve reproducibility. Therefore, Lee [59] grew well dispersed p-type NiS nanoparticles on the surface of a highly aligned n-type TiO_2_ film to obtain the NiS-sensitized TiO_2_ films. The band gaps of two components were estimated by wavelength relation. Some inferences can be drawn when considering the results of both the ultraviolet and visible spectra. It indicates that more electrons are subpoenaed from the short-*E*g NiS and transferred to TiO_2_ conduction band. The spectra results reconfirmed the electron contribution of the NiS and the design of a catalyst that produced 3.77-fold CH_4_ compared to the TiO_2_ film.

#### 3.1.5. Summary

Overall, some of the photocatalytic systems that use TiO_2_ is presented in Table 1.

### 3.2. WO_3_-Based Photocatalysts

Tungsten-based oxides (WO_3_) have been extensively studied in recent decades and various morphologies have been presented. In the WO_3_ structure, the crystal in the stoichiometric ratio is connected with a twisted WO_6_ octahedra to form a perovskite crystal structure. It has monoclinic, orthorhombic and hexagonal crystal forms. At the same time, the oxygen lattice can be lost easily, resulting in oxygen vacancies and unsaturated, coordinated W atoms. Therefore, tungsten oxide has many non-stoichiometric compounds, such as WO_2.72_, WO_2.8_, WO_2.83_ and WO_2.9_. Of these, WO_3_ is the most common and has been widely studied as a typical photocatalytic water oxidation semiconductor material. WO_3_ is a typical narrow-band gap indirect semiconductor with a forbidden band width of 2.6–2.8 eV, which can absorb part of the visible light [64]. In addition, WO_3_ is a research hotspot in the field of photoelectrochemical water splitting because of its high carrier mobility, stability in acidic electrolytes and resistance to photocorrosion.

#### 3.2.1. Morphology Control

Bi_2_WO_6_ is one of the tungsten-based materials that belongs to Aurivillius crystal oxides. Its crystal has an orthorhombic system, and its narrow band gap (2.7–2.9 eV) structure allows it to meet the response absorption of visible light. Moreover, its stable structure and eco-friendly properties have attracted many scientific researchers to study it. Since the valence band of Bi_2_WO_6_ is composed of O_2p_ and Bi_6p_, and the conduction band is composed of W_5d_-assisted Bi6p orbitals, the VB energy levels can be dispersed broadly. By employing the Kirkendall effect in ion exchange and BiOBr precursor, Huang et al. [65] prepared a bowl-shaped Bi_2_WO_6_ HMS material. Based on the large specific surface area of the material, its adsorption capacity for CO_2_ reaches 12.7 mg g^−1^ at room temperature and pressure. The material adsorbs a large number of HCO_3_^−^ and CO_3_^2−^ species on the surface during the reaction, which makes the catalytic reaction easier. The Bi_2_WO_6_ HMS thus has a high catalytic activity, and the methanol yield is 25 times higher than that of the Bi_2_WO_6_ SSR.

Iron phthalocyanine FePc is neatly assembled on porous WO_3_ under induction and coupled with surface atoms by H-bonding [66]. The optimized FePc/porous WO_3_ nanocomposites exhibit enhanced CO_2_ photoreduction activity, which is attributed to the synergistic effects of a high specific surface area, a better charge separation and proper central metal cation. A series of mesoporous WO_3_ with interconnected networks were synthesized by the silica KIT-6 hard template method [67], which became oxygen-deficient after hydrogenation treatment. Both the ordered porous structure and oxygen vacancies contributed to the increased yield of CH_4_ and CH_3_OH.

**Figure 10:**
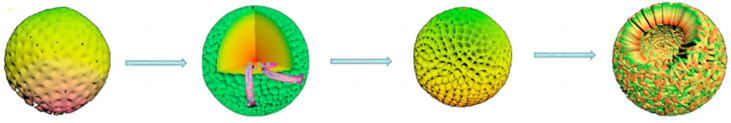
Schematic diagram of the HNWM formation process [68].

WO_3_ with a hollow nest morphology with hierarchical micro/nanostructures (HNWMs) was synthesized [68] by the one-step hydrothermal method (Figure 10), with a particle diameter of about 2.5 μm. The 2D nanosheets, which have an average thickness of 30–40 nm, were assembled to build a distinctive hollow nest structure with a good stability and reusability under visible light. Hao et al. [69] prepared core–shell heterojunctions of two-dimensional lamellar WO_3_/CuWO_4_ by the in situ method. After the modification of amorphous Co-Pi co-catalyst, the photoanode of ternary homogeneous core–shell structure exhibited a high photocurrent of 1.4 mA/cm^2^ at 1.23 V/RHE, which was 6.67 and 1.75 times higher than that of the pristine WO_3_ and 2D homogeneous heterojunction. Ren et al. [70] synthesized unique flower-like Bi_2_WO_6_/BiOBr catalysts by the simple one-step solvothermal method, and showed that the photocatalytic activity of the composites was significantly enhanced due to the construction of type II heterojunctions. The presence of Br source enhanced the light absorption and improved charge-carrying spatial transfer and separation.

Ti atoms in ultrathin Ti-doped WO_3_ nanosheets promoted the charge transfer [71], as they accelerate the generation of key intermediates COOH*, which was revealed by in situ characterization. Furthermore, Gibbs free energy calculations were calculated to verify that ion doping can reduce the CO_2_ activation energy barrier and CH_3_OH desorption energy barrier by 0.22 eV and 0.42 eV, respectively, thus promoting the formation of CH_3_OH. The ultrathin Ti-WO_3_ nanosheets showed an excellent CH_3_OH yield of 16.8 μmol g ^−1^ h ^−1^. Two-dimensional bilayered WO_3_@CoWO_4_ were prepared [72] via a facile interface-induced synthesis method. The optical energy conversion efficiency can be improved by both p–n heterojunctions and interfacial oxygen vacancies. The narrow band gap of the WO_3_@CoWO_4_ heterojunction was proved by DFT calculations and some characterizations, which allows a better visible light absorption. A tree-like WO_3_ film was prepared [73] by the hydrothermal process, which has a large specific surface area. The WO_3_ product was a unity of hexagonal/monoclinic crystals, which contained W^5+^ defects and oxygen vacancies. The products were further subjected to a mild reduction solution at the lower temperature of 333 K to introduce more defects. It turns out that the intermediate state induced by defects diminished the band gap. A reasonable amount of defects benefits the photocatalytic activity of WO_3_, while too many defects impair its catalytic capacity. The performance of the treated WO_3_ films increased 2.1 times in 48 h compared to that of the annealed WO_3_ samples.

##### Preferentially Exposed Facets

According to studies, infrared (IR) light makes up nearly 50% of solar energy, and it is challenging to make use of the majority of the light. Liang et al. [74] fabricated 2D ultrathin WO_3_ with an intermediate band gap. They achieved the first complete decomposition of CO_2_ driven by infrared light without the addition of sacrificial agents. Theoretical calculations indicated that the generation of the intermediate energy band resulted from the critical density of the generated oxygen vacancies, which has also been verified by synchrotron valence band spectroscopy, photoluminescence spectroscopy, ultraviolet-visible-near-infrared spectroscopy and synchrotron infrared reflection spectroscopy. The results showed that the WO_3_ atomic layer containing oxygen vacancies can achieve the complete decomposition of CO_2_ and generate CO and O_2_ under infrared light.

Microscopic WO_3_ nanocrystals were formed by Chen et al. [75] through solid–liquid phase arc discharge in an aqueous solution. Then, they synthesized ultrathin single-crystal WO_3_ nanosheets via a laterally oriented attachment method. The quantization effect of this nanostructure altered the bandgap width of WO_3_ nanosheets, enabling the semiconductor to exhibit a high performance at a wide range of nanometer sizes. It is beneficial to control the activity and selectivity of the photoconverted CO_2_ products. As a consequence, WO_3_ with a strong visible light response has enormous potential in the field of photocatalytic CO_2_ reduction.

Bi_2_WO_6_ has a positive conduction band potential, which is not enough to excite and reduce CO_2_ molecules, thus limiting its application in the photocatalytic reduction of CO_2_. Therefore, researchers have been devoted to modifying the surface of Bi_2_WO_6_ to improve its photocatalytic activity in order to obtain a high-efficiency CO_2_ reduction ability. Zhou et al. [76] successfully prepared Bi_2_WO_6_ nanosheets with a monolayered structure by introducing the surfactant CTAB (hexadecyltrimethylammonium bromide) into the precursor solution. A large number of unsaturated Bi atoms were produced, which provided sufficient active sites for photocatalytic reactions. Bi_2_WO_6_ is composed of a [BiO]^+^-[WO4]^2−^-[BiO]^+^ sandwiched layer structure. Under irradiation, holes and electrons are generated in different [BiO]^+^ layers. This structure promotes the spatial separation of photogenerated electron–holes and greatly reduces the carrier recombination rate of the monolayer Bi_2_WO_6_ material.

With the assistance of an oil-based primary amine (C_18_H_37_N) surfactant, Zhou et al. [77] conducted a hydrothermal reaction at 200 °C for 20 h to prepare an ultra-thin and uniform Bi_2_WO_6_ nanosheet. The material has a strong response under visible light, and its forbidden band width was about 2.44 eV through theoretical calculations, with a conduction band potential of −0.31 e V. Then, CO_2_ could be easily reduced to CH_4_, and its yield was 20 times higher than that of SSR Bi_2_WO_6_. The light-absorbing capacity will decrease if the nanosheets are too thin because of the quantum size effect. Therefore, scholars need to consider roundly when designing fresh material [78].

##### DOM Structure W-Based Materials

Unexpectedly, it was found that the resistance of 3DOM-WO_3_(270) and the Ag_3_PO_4_ electron absorption band were comparable. By depositing Ag_3_PO_4_ nanoparticles in the micropores of 3DOM-WO_3_, Chang et al. [79] achieved a higher photocatalytic activity and more efficient light harvesting at the wavelengths of 460–550 nm. A Z-scheme g-C_3_N_4_/3DOM-WO_3_ catalyst designed by Tang et al. [80] also has a high CO_2_ photoreduction activity. The separation way of the photogenerated electron–hole pairs determined its Z structure, and 3DOM framework heightened the light collecting efficiency. Therefore, an excellent photocatalyst exhibited a high CO evolution rate of 48.7 μmol g^−1^ h ^−1^.

#### 3.2.2. Heterojunction

**Figure 11:**
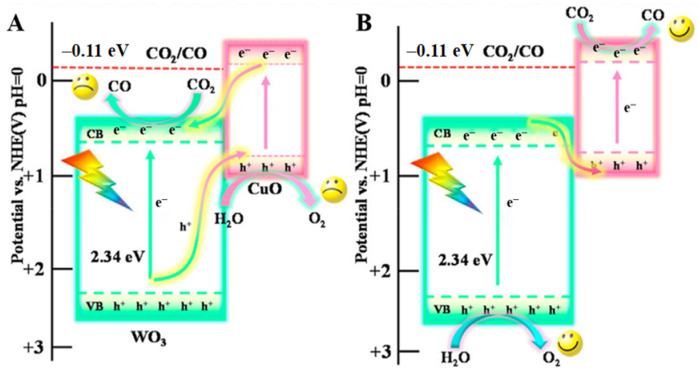
The proposed charge transfer mechanisms: (**A**) Ⅱ-scheme and (**B**) Z-scheme for CuO/WO_3_ [81].

Quantum dot composite: CuO quantum dots (QDs) were combined with WO_3_ nanosheets by a self-assembly method and the diameter of 6%CuO QDs/WO_3_ NSs was mainly located at 1.6 nm [81]. The bandgap energy of CuO/WO_3_ fell in 2.28 eV and the complex catalyst possessed a lower resistance for charge carrier transfer that showed in UV-vis DRS and EIS analysis. Due to the low CB position, CO cannot be obtained when using pure WO_3_. However, the photogenerated electrons gathered in the WO_3_ CB position was able to reach the CuO VB position when the Z-scheme (Figure 11) was formed by intimate heterojunctions. At the same time, the reduction reaction that transformed CO_2_ into CO occurred at the CuO CB position. The high yield rate of about 1.58 mmol g^−1^ h^−1^ also benefited from a longer fluorescence lifetime, and reduced the overlap of electron pore pairs.

Perovskite composite: For WO_3_, the negative potential energy of the sheet shape (−31.5 mV) was lower than that of the rod (−21.0 mV). In addition to its potential advantages, S–WO_3_ offers a higher specific surface area. Defects occurred because of the exposed interior atoms in nanosheets surface, which promoted the CO_2_ adsorption. Positively charged perovskite cesium lead tribromide (CsPbBr_3_, CPB) with a long electrically diffused length was selected to be combined with S–WO_3_, affording a high field rate of CO and CH_4._ Zhang and others [82] employed a three-dimensional hydrophobic porous melamine foam to support the mixture, not only to protect the CPB from dissolution but also to reduce toxic Pb^2+^.

#### 3.2.3. Ion Doping

Molybdenum with a similar ionic size was chosen to dope the WO_3_ as a low-valence metal species. The W^5+^/W^6+^ ratio of the catalyst was increased, making it easier to exchange electrons with reactants. The conductivity of protons was enhanced by the presence of hydrogen bronze, which originated from a chemical reaction between WO_3_ and Brønsted protons and excess electrons in their lattices. Wang et al. [83] prepared molybdenum-doped WO_3_·0.33 H_2_O by the hydrothermal method. The E_cb_ and E_vb_ energy were both higher at 3%Mo-WO than WO_3_. After 20 min of the FTIR spectra, rare intermediate CO^2−^ was observed, verifying the activation of CO_2_, which was more common in low-valent meta species. The content of potassium hydroxide in an aqueous solution obtained by photocatalytic water oxidation was higher and the CH_4_ yield was 4.2 times higher than WO_3_.

#### 3.2.4. Summary

To date, Bi_2_WO_6_ semiconductor photocatalytic materials have made great progress in the field of environmental management. Some photocatalytic systems using WO_3_-based materials in CO_2_ conversion are listed in Table 2.

### 3.3. ZnO-Based Photocatalysts

ZnO, a common metal oxide, is a n-type semiconductor with an *E*g value of 3.37 eV. It is a kind of amphoteric oxide that has the advantages of nontoxic harmlessness, low cost, abundant reserves, convenient preparation, low dielectric constant and low optical coupling rate. ZnO has three main lattice structures: wurtzite structure, zinc-blended structure and tetragonal rock salt structure. The wurtzite structure is considered the most stable and common structure in nature. It is a kind of hexagonal crystal, in which the O and Zn atoms are aligned with the hexagonal density stacking. The photodegradation process of ZnO is similar to TiO_2_ and it has been widely used in photocatalysts, solar cells and conducting materials.

#### 3.3.1. Morphology Control

**Figure 12:**
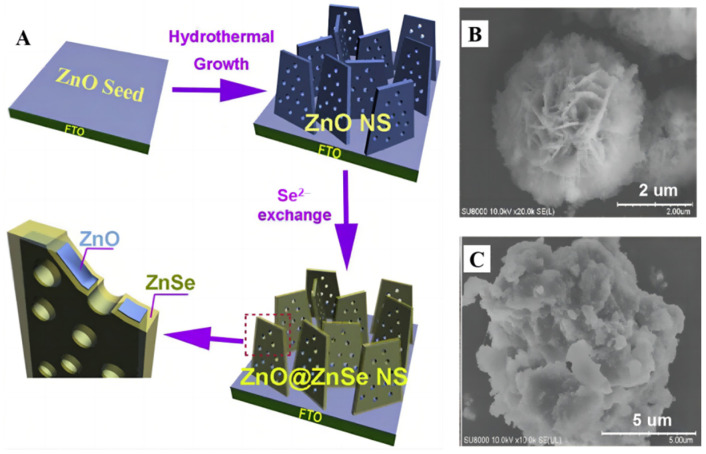
(**A**) Schematic illustration of the fabrication process of ZnO@ZnSe photocathode [88]. (**B**) ZnO/ZnS (**C**) ZnO/ZnS/g-C_3_N_4_ SEM images [91].

The 3nm Pt particles were uniformly dispersed over ZnS@ZnO with a mesoporous heterostructure [90] and more CH_3_OH was obtained. Reactant charge carriers entered the pore channels of the porous heterozygous layer, thus reducing the likelihood of flow resistance and electron–hole recombination. The S-scheme photocatalyst delivered a high CH_3_OH formation rate of 81.1 μmol g^−1^ h^−1^, which is roughly 40 and 20 times larger than that of bare ZnO (3.72 μmol g^−1^ h^−1^) and ZnO–ZnS (4.15 μmol g^−1^ h^−1^). On the other hand, a porous ZnO@ZnSe core/shell nanosheet array material (Figure 12A) was prepared in a controlled manner [91]. The final n-type semiconductor composites had a proper negative CB band edge. In comparison to ZnO or ZnSe, more pairs of electron–holes were formed under visible light. Electrons tend to land on ZnO, which is aimed at methanol production. Mei et al. prepared a ZnO microsphere with different numbers of shells [92] and the photoelectric performance of ZnO was optimal when the number of shells reached three.

It is challenging to coat uniform 2D g-C_3_N_4_ nanofilm on the surface of 3D materials because of the difficulty in exfoliation process. Thus, Wang et al. [93] proposed an electrostatic method and incorporated g-C_3_N_4_ nanofilm with porous ZnO nanospheres that has a strong interaction. The heterojunction was then anchored on 3D graphene aerogels (GAs). The compound g-C_3_N_4_/ZnO/GA has extraordinary stability, maintaining a high CO_2_ conversion rate, which is 92% of its original activity after 100 h.

ZnO/ZnS nanoflowers (Figure 12B) were combined with g-C_3_N_4_ nanosheets (Figure 12C) to construct a double Z-scheme structure [94]. ZnO/ZnS nanoflowers provide a large specific surface area and g-C_3_N_4_ helps to absorb more photons under solar light irradiation. Optimized interfacial charge transfer dynamics in ternary heterostructure can be characterized by photocurrent measurements. As a result, the formation rate of H_2_ product over the novel double Z-scheme mixture increases to 301 μmol g^−1^ h^−1^ on water splitting.

Hierarchical CuO/ZnO nanocomposites with p-n heterojunction were prepared [95] by the modified hydrothermal method. The photocatalysts are found to converse CO_2_ to methanol in aqueous solution containing dimethylformamide (DMF) and triethylamine (TEA) as electron donor under visible light irradiation. ZnO/NiO porous hollow spheres with sheet-like subunits were obtained [96] by calcination of Ni-Zn MOFs. Numerous p-n heterojunctions with n-type ZnO and p-type nickel monoxide were formulated in mixed ZnO/NiO. The porous hollow structure with the large specific surface area can increase the absorption capacity of CO_2_ and light.

Based on the vapor to solid mechanism, a novel ternary Ag/CeO_2_/ZnO nanocomposite [97] was synthesized by the facile thermal decomposition method. Oxygen vacancies introduced by line structure contributied to a narrow band gap of 2.66 eV, and this was further confirmed by DRS characterization. After the preparation of the ZnO/TiO_2_ nano-tree arrays, Ag_2_S and ZnS were synthesized to modify the nano-tree arrays by cation exchange methods [98]. The core-shell structure of ZnO@ZnS prevented the decomposition of ZnO, and the modification of Ag_2_S reduced the *E*g value of the composite and promoted the red-shifted of light absorption.

#### 3.3.2. 3DOM Structure Zn-Based Materials

**Figure 13:**
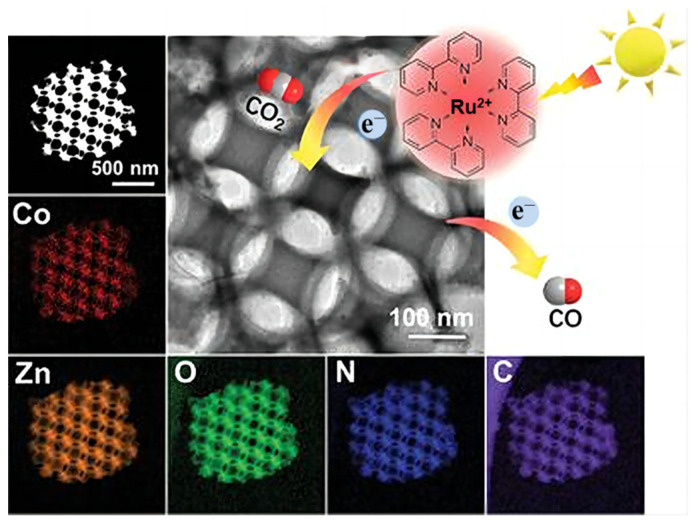
HAADF-STEM image of CoO_x_/N-C-ZnO and related elemental mapping images [99].

Wang et al. [99] published metal-organic-framework-derived 3DOM N-C doped ZnO (Figure 13) for efficient CO_2_ reduction. The ultra-tiny CoO_x_ clusters were anchored on the surface of catalyst and no Co-Co peak was found in CoO_x_/N-C-ZnO. The charge transfer rate was jacked up by ion doping and the recombination of electron-hole pairs was tamed because of the CoO_x_ clusters. Furthermore, CoO_x_ on the orderly connected channels can act as an electron trap to capture electrons, which makes a contribution to photoreaction efficiency. The density theory calculations (DFT) was also used to detect the CO_2_ adsorption ability, and CoO_x_/N-C-ZnO exhibited the most negative CO_2_ binding energy due to improved electron structure of adsorption site.

#### 3.3.3. Heterojunction

Recently, zeolitic framework (ZF) composite fabricated by the microwave-hydrothermal synthesis method (MWH) has attracted attention, which can provide a fast heating-speed and produce morphologically uniform samples. With biodegradable template, the zeolitic framework (ZF) was synthesized via MWH method from volcanic ashes. The NaAlSiO_4_ (NAS) framework was composed of 50 nm circular channels and has a large surface area. Hip’olito et al. [18] embedded ZnO/CuO hybrid structure in the NAS channels, resulting in a ternary composite. The synergistic effect among ZnO, CuO and ZF support accelerated the photocatalytic process of water splitting and CO_2_ reduction, which offers higher H_2_ and HCOOH evolution rate.

**Figure 14:**
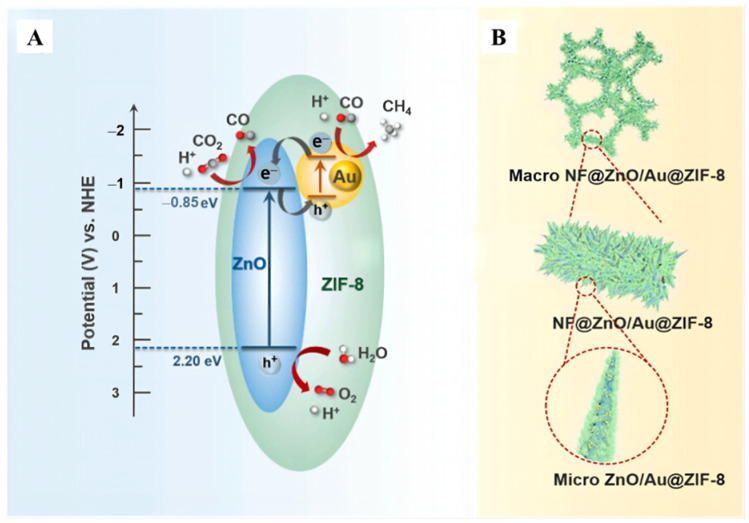
(**A**) Illustration of the band structure and charge transfer process of NF@ZnO/Au@ZIF-8 catalyst under UV− vis light. (**B**) The structural diagram the monolithic NF@ZnO/Au@ZIF-8 photocatalyst [100].

The imidazole framework-8 (ZIF-8) molecular as a widely used zeolite (ZIF) composite, has an excellent competence of absorbing CO_2_, which is apt for CO_2_ converting. Selective-breathing effect was wielded to boost CO_2_ conversion efficiency through monolithic NF@ZnO/Au@ZIF-8 (Figure 14A) catalyst [100]. Au particles were loaded on ZnO nanorods that grown on Ni foam (NF), the mixture was immersed in methylimidazole solution (Figure 14B). Based on the results of electrochemical impedance spectroscopy (EIS) Nyquist plots, the alternating magnetic field was introduced to create magnetic heat, which leading to increased carrier density and improved photocatalytic performance. It is found that the selectivity of CH_4_ cheeringly achieved 89% from 61% under photo-thermal-magnetic coupling effect.

#### 3.3.4. Ion Doping

Many metal elements have been doped in ZnO to minish its band gap, such as Sb, Cu and Mn, among them Co distinguished itself because of the similar ion radius. In addition, the conversion of Co^3+^ and Co^2+^ results in oxygen vacancies, which greatly enhances photocatalytic efficiency. Xie et al. [101] introduced Co^3+^ with different mole ratio to ZnO microspheres precursor (including Zn^2+^, urea, and PVP), and the introduction of Co^3+^ did not disrupt lattice structure as seen in the XRD model. It was revealed that the presence of both Co^2+^ and Co^3+^ in Co-ZnO from high-resolution spectrum, and Zn_2p_ exhibited higher binding energy due to Zn^2+^ charge transfer in 7% Co-ZnO. As the ratio of Co/Zn increased, the conversion from Co^2+^ to Co^3+^ decreased, and the light response range gradually expanded. The results showed that the electrochemical impedance of 7% Co-ZnO sample was the lowest band gap of 2.56 eV.

#### 3.3.5. Summary

Great efforts have been made to design catalysts for CO_2_ reduction on ZnO and the relevant research data are summarized in Table 3.

### 3.4. Cu_2_O-Based Photocatalysts

Cuprous oxide (Cu_2_O) is a potential p-type semiconductor with a wide visible-light response range and high photo-electric conversion efficiency (18%) [106], and it displays attractive prospects in solar energy conversion and heterogeneous photocatalysis. Although Cu_2_O possesses many excellent properties, photocorrosion and the rapid recombination of e^−^/h^+^ pairs affect its activity and limit its application. The photocorrosion is believed to occur in two ways: (1) self-reduction caused by generated electrons and (2) self-oxidation caused by the generated holes.

Self-reducing photocorrosion: Cu_2_O + H_2_O + 2e^−^ → 2Cu + 2OH^−^ (10)

Self-oxidative photocorrosion: Cu_2_O + 2OH^−^ + 2h^+^ → 2CuO + H_2_O (11)

Therefore, developing Cu-based catalysts with excellent activity, selectivity and stability has become the research hotspot in the area of the photocatalytic reduction of CO_2_. Many successful attempts have been made to improve the photostability and photocatalytic performance of Cu_2_O. In general, most studies focus on enhancing the charge transfer from Cu_2_O to reactants or cocatalysts to prevent charges from accumulating within the particles. A series of methods for improving the performance of Cu_2_O are discussed in detail in what follows.

#### 3.4.1. Morphology Control

A branch-like Cd_x_Zn_1-x_Se nanostructure was obtained [107] by the cation-exchange method, which was then mixed with Cu_2_O@Cu to form heterojunctions. Selenium (Se) vacancies were created during the ion exchange process and the crystal growth was limited due to the additive diethylenetriamine (DETA), leading to insufficient coordination of the surface atoms, which then become active adsorption sites. Highly hierarchical branching-like structures assembled by one-dimensional structural materials not only facilitate electron accumulation at their tips but also increase the light-accepting area, and characterization results show that branching structures can effectively absorb visible light. Cd_0.7_Zn_0.3_Se/Cu_2_O@Cu step-scheme heterojunction exhibited a CO release yield of 50.5 μmol g^−1^ h^−1^.

**Figure 15:**
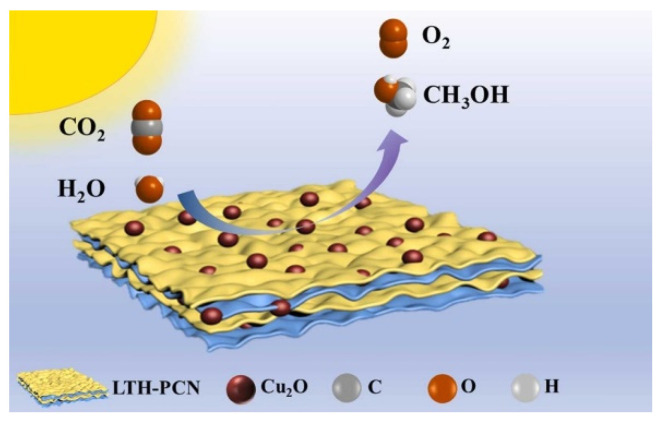
Ultrafine U-Cu_2_O nanoclusters anchored on the photosensitizing PCN support [108].

Ultrafine cuprous oxide U-Cu_2_O (<3 nm) was grown on the polymeric carbon nitride (PCN) (Figure 15) by the in situ method [108]. PCN has a narrow band gap of 2.7 eV that can capture visible light. Both ultrafine nanoclusters and Z-scheme heterojunction can protect U-Cu_2_O from degradation. The photocatalyst U-Cu_2_O-LTH@PCN has high stability, maintaining more than 95% activity after five cycles of testing, while bare Cu_2_O grades completely within three cycles. A large number of heterojunctions were formed by U-Cu_2_O particles and lamellar PCN, expediting the electron transfer efficiency. The product can convert CO_2_ to methanol with water vapor under light irradiation at the high yield of 73.46 μmol g^−1^ h^−1^. Ultrathin Ti_3_C_2_MXene with a high fraction of coordinated unsaturated surface sites was fabricated by Zhang et al. [109]. Via the HF etching method, different amounts of Cu_2_O were combined with Ti_3_C_2_ nanosheets under the hydrothermal condition. The unique hexagram morphology of Cu_2_O, the 2D layer structure and excellent conductivity of Ti_3_C_2_T_x_ nanosheets and the synergistic effect between the two composites promote the improvement of photoactivity. Zhang et al. reported the bifunctional catalyst of Cu_2_O@Fe_2_O_3_. Cu_2_O nanoparticles coated with an Fe_2_O_3_@carbon cloth electrode were used for both overall water splitting and CO_2_ photoreduction [110].

##### Preferentially Exposed Facets

**Figure 16:**
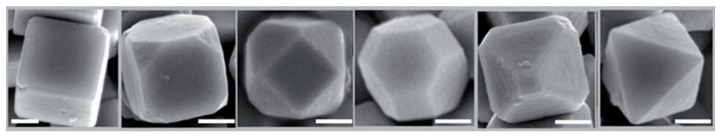
FESEM images of the Cu_2_O polyhedrons with different volume ratios of (100) to (111) [111].

Cu_2_O is an ideal compound to study the influence of electron-related effects. The rare occurrence of the O-Cu-O 180^o^ linear coordination of Cu_2_O makes its (111), (100) and (110) facets chemically active. Zhang et al. [111] successfully achieved the morphology control of Cu_2_O nanocrystals by utilizing the selective surface stabilization of PVP on the (111) plane of Cu_2_O. With different amounts of PVP, the surface area ratio of (111) to (100) was subtly tuned, which resulted in the shape evolution of the system and various Cu_2_O structures (Figure 16). The detailed modification mechanism was elucidated from the structural and kinetic perspectives.

Octahedral copper oxide that exposes the (111) crystal faces was decorated with low Fermi energy Ag nanoparticles. After coating with rGO, the ternary heterojunction catalyst [112] exhibits selective photocatalytic superiority towards CH_4_. The CO* radicals can be characterized via DRIFT spectra and DFT calculations, which is the key intermediate for the conversion of CO_2_ to CH_4_. Two types of MoS_2_ (p-type and n-type) and two shapes of Cu_2_O (cubic and octahedron) were synthesized and combined with each other [113], and the compositions possessed different electronic and structural properties. The heterostructures formed by the p-type MoS_2_ with intrinsic conductivity had a higher photocatalytic activity, and the methanol production yield was as high as 76 μmol g^−1^ h^−1^. Both Z-type and ii-type charge transfer mechanisms were built using an n-type MoS_2_ mixture. For Cu_2_O, the cubes of the exposed (100) crystal plane with a higher binding affinity with MoS_2_ transferred electrons more efficiently and produced methanol at a higher rate.

A 3D porous Cu was produced by electrodeposition method [114], being transformed into CuO_2_ after following high-temperature annealing. Three-dimensional Cu_2_O delivers a 24-fold production of CO compared with the unremarkable and non-porous Cu. Additionally, more CO_2_ accumulated and took reactions in the hollow space of 3D Cu_2_O to form C_2_ products.

##### 3DOM Structure Cu-Based Materials

**Figure 17:**
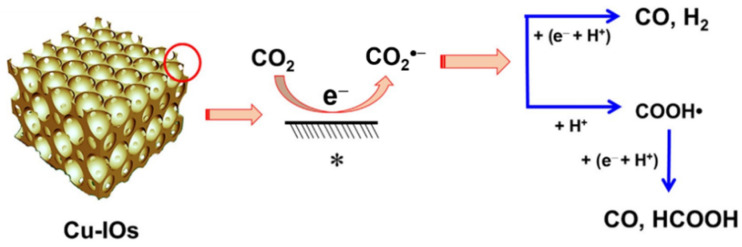
Proposed mechanism for CO_2_ reduction to CO and HCOOH on Cu_2_O-derived Cu-IOs (The symbol “*” represents the surface.) [116].

The 3DOM Cu_2_O structure was luckily obtained [115] via polystyrene crystal templates. Under the contrived “sunlight” irradiation, incident light was reflected and absorbed around and around again. In the ultra-visible absorption spectra (350 to 800 nm), Cu_2_O with large orifices absorbs more photons than bulk samples, making it more advisable for solar applications. 3DOM Cu_2_O was prepared [116] by the electrochemical method to reduce CO_2_, and its Faraday efficiency was five times higher than that of Cu film. The CO_2_·^−^ intermediate in 3DOM channels is more stable and leads to the possibility of forming CO and HCOOH products (Figure 17). We look forward to the applications of inverse opal Cu_2_O in photocatalysis.

#### 3.4.2. Heterojunction

Liu et al. [117] reported a facile solution and chemistry route to synthesize rGO-incorporated crystal Cu_2_O with various facets as visible-light-active photocatalysts for CO_2_ reduction. The enhanced activity was attributed to the formation of the heterojunction and the existence of rGO as the electron transport mediator. M. Flores et al. [118] adopted the microwave-hydrothermal method to couple the powders of Mg(OH)_2_, CuO and Cu_2_O. The synthesis method allowed a sufficient interaction between Mg(OH)_2_/CuO and Cu_2_O without inhibiting the gas adsorption capacity of Mg(OH)_2_. They found that the presence of Cu_2_O favored the selectivity towards CH_3_OH production because a higher Cu^+^ concentration led to better selectivity. Niwesh et al. [119] reported the formation of a p–n heterojunction between Cu_2_O and the SnS_2_/SnO_2_ nanocomposite that offered favorable reductive potentials and high stability, mainly owing to their intimate interfacial contact. In the absence of a sacrificial agent, the generation rate of NH_4_^+^ was 66.35 μmol g^−1^ h^−1^ for Cu_2_O/SnS_2_/SnO_2_, which is 1.9-fold higher than that of SnS_2_/SnO_2_. However, the work of Trang et al. also demonstrated the instability and photo-oxidation of Cu_2_O heterojunctions. Generally, most p–n heterojunctions are found to reduce the redox capacity of photogenerated charges. This is especially evident for Cu_2_O p–n type heterojunctions, as CuO is excessively formed on the surface of Cu_2_O under continued illumination, so there are recombination problems at the heterojunction interface. The construction of Z-scheme heterojunctions overcomes the limitations of p–n heterojunctions, namely the reduction in redox potential and charged carrier recombination at the p–n heterojunction interface. In a Z-type heterojunction, the redox potential can be maintained under the premise of high photo-induced electron transport rate.

**Figure 18:**
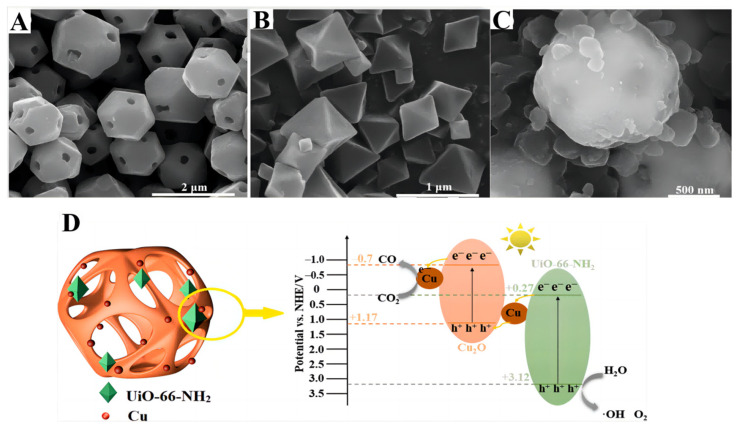
(**A**–**C**) SEM results of Cu_2_O, UiO-66-NH_2_ and U/C/Cu-0.39. (**D**) Illustration of the photocatalytic CO_2_ reduction in U/C/Cu-0.39 [122].

Zhang et al. [120] synthesized coal-based CNPs with an sp^2^ carbon and multilayer graphene lattice structure, and loaded them onto the surface of Cu_2_O nanoparticles prepared by the in situ reduction of copper chloride. The rapid recombination of electron–hole pairs was suppressed by the introduction of CNPs. The energy gradient formed on the surface of Cu_2_O/CNPs facilitates the effective separation of electron–hole pairs for CO_2_ reduction, improving the photocatalytic activity. Atomically dispersed In-Cu bimetallic catalysts were prepared [121] by the in situ pyrolysis method, in which carbon nitride acted as a carrier. The light-harvesting and charge separation efficiency were enhanced by regulating the loading amount of Cu and In, and the supreme generation rate of the photoreduction of CO_2_ to ethanol reached 28.5 μmol g^−1^ h^−1^ with 92% selectivity. The DFT calculations showed that the introduction of an In atom in copper can accelerate electron transfer from carbon nitride to metal, improve the charge separation efficiency and increase the electron density of copper active sites. The presence of In–Cu sites exerted a synergistic effect, which could promote C–C coupling, lower the energy barrier of *COCO generation and increase ethanol yield. Zhao et al. [122] reported the indirect Z-scheme heterojunction of UiO-66-NH_2_/Cu_2_O/Cu, which achieved a high CO_2_ photocatalytic conversion to CO. The SEM results of Cu_2_O, UiO-66-NH_2_ and U/C/Cu-0.39 are shown in Figure 18A–C. In this catalytic system, UiO-66-NH_2_ slowed down the photo-corrosion rate of Cu_2_O and increased the CO_2_ adsorption capacity (Figure 18D).

#### 3.4.3. Summary

In conclusion, photochemical methods offer the opportunity to modulate the persistence and selectivity of Cu-based catalysts photoreduction to value-added compounds. The most recent photocatalytic CO_2_ reduction outcomes of Cu-based materials are listed in Table 4.

### 3.5. CeO_2_-Based Photocatalysts

Cerium oxide (CeO_2_) has an octahedral face-centered cubic fluorite structure, in which the coordination numbers of Ce and O are 8 and 4. When reduced at a high temperature, it can be converted to nonstoichiometric CeO_2−x_ (0 < x < 0.5). Notably, CeO_2−x_ maintains a fluorite crystal structure and forms oxygen vacancies after losing a certain amount of oxygen. CeO_2−X_ materials with different Ce/O ratios were also obtained in different conditions and it could be reconverted to CeO_2_ again if it returned to an oxidizing environment. Because of the unique electrical structure, cerium oxide (CeO_2_) is famous for the conversion sates between Ce^4+^ and Ce^3+^, which have been studied as oxygen storage catalytic materials and solid oxide full cells by many scholars [124,125]. In summary, CeO_2_ is a rare-earth metal oxide with a good photochemical stability, low cost and environment friendly characteristics. It is an important n-type semiconductor with a wide bandgap, and credible photocatalysts have been designed to reduce CO_2_ and degrade pollutants [126].

#### 3.5.1. Morphology Control

Yb-, Er-doped CeO_2_ hollow nanotubes were synthesized [127] using silver nanowires coated with silica, and the products had a narrower band gap of 2.8 eV. The core–shell structured CeO_2_ was converted into mesoporous hollow spheres by the Ostwald ripening method in the presence of urea and hydrogen peroxide [128]. CeO_2_ nanocages can be fabricated by mixing (NH_4_)_2_Ce(NO_3_)_2_ with templates of Cu_2_O nanocubes [129], in which Cu_2_O is finally sacrificed. The photocatalytic results [130] indicated that CeO_2_ nanocages exhibit higher activity than hollow spheres.

##### Preferentially Exposed Facets

**Figure 19:**
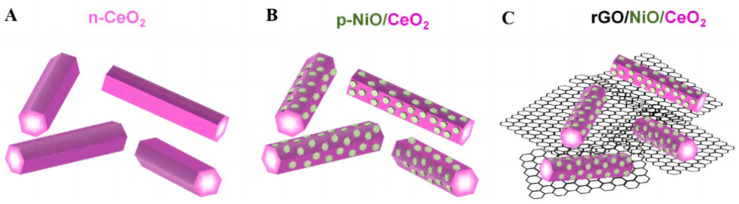
The structural diagrams of (**A**) n-CeO_2_ nanorods, (**B**) p-NiO/CeO_2_ composite and (**C**) NiO/CeO_2_/rGO hybrid composite [132].

It was found that molecular CO_2_ can be distorted and participate in reactions at a low energy on the CeO_2_ surface [131]. A p-type NiO material was designed to modify the rod-like CeO_2_ nanostructure [132], allowing electrons and holes to migrate to opposite directions. They then operated the Mott–Schottky test, which showed a typical p–n junction. The presence of hexagon-shaped NiO plates broadened the range of light responses, which can be verified in the UV-Vis absorption spectra. Graphene oxide (rGO) was introduced as a “network” of for photoreduction electron transportation (Figure 19A–C). The impedance can be seen in the EIS Nyquist plot, which shows that the NiO/CeO_2_/rGO achieved the minimum value. The HCHO production rate of the ultimate catalyst was 421 μmol g^−1^h^−1^ with the synergy of several favorable factors. It is worth mentioning that a range of in situ techniques have been used to detect oxygen vacancies, structural changes, free radicals and formate on the surface of CeO_2_.

##### Macroporous

Mesoporous N-doped CeO_2_(NMCe), a relatively ordered intermediate structure with enhanced CO_2_-capturing capability, was prepared without any convoluted procedures or expensive equipment [124]. In the Roman spectrum, the bands from 550 to 650 cm^−1^, which are closely related to oxygen vacancy, were more salient than the MCe band. In addition, N-doped porous CeO_2_ has a higher CO_2_ absorption capacity than porous CeO_2_. All the above results conformed to the photoluminescence spectrum (PL) analysis, and the reduction gross yield of CO and CH_4_ was 3.5 times higher than that of OMCe.

With the help of a suitable crosslinked and pyrogenic solvent, doped CeO_2_ was uniformly fixed on transparent polymers by the in situ polymerization pathway [133]. By increasing the Ca/Ce molar ratio (wt.% < 20%), no peaks relating to CaO were observed in the p-XRD of samples and the original diffraction peak intensity became more strident due to the foreign ion’s minuscule radius (Ca^2+^ 0.1 nm, Ce^4+^ 0.184 nm). The specific surface areas of three catalysts (CeO_2_, (20: 80) CaO/CeO_2_ and CaO/CeO_2_ NC-dispersed polymers as a whole) were 28.4, 58.3 and 224.7 m^2^ g^−1^, respectively, and heterojunction nanocomposites had the highest photocatalytic efficiency.

##### 3DOM Structure Ce-Based Materials

Under the protection of poly alcohol, Zhang et al. [134] synthesized 3DOM CeO_2_ that was loaded with Au–Pd alloys. 3DOM CeO_2_ photocatalytic materials are expected to emerge in the field of CO_2_ emission reduction, which could open up more possibilities for the development of super-catalysts.

#### 3.5.2. Heterojunction

Researchers tried to combine CeO_2_ with g-C_3_N_4_, which is popular for its energy bands and chemical stability. Through hard work, a three-dimensional porous g-C_3_N_4_(3DCN) was achieved, with the advantages of multi-channel structure. To accommodate more electrons and heighten the density of photoelectric currents, Zhao et al. [125] loaded Pt nanoparticles (5–6 nm) on CeO_2_/3DCN using photodeposition techniques that require UV lamp radiation. The photoreduction rate gradually increased as the CeO_2_ amount rose in the range of 15~45% and the yield rates of 4.69 and 3.03 μmol·h^−1^·g^−1^ for CO and CH_4_ were achieved, respectively, after decorating with Pt crystalline grains.

Under mild reaction conditions, carbon-doped hexagonal boron nitride (h-BN), known as boron carbon nitride (BCN), can reduce more carbon dioxide after dispersing cerium oxides on it. In the BCN/CeO_2_ heterostructure [135], the N-O-Ce bond was formed by thermal precipitation method. It is of interest that the proportion of Ce^3+^/(Ce^3+^ + Ce^4+^) that influences electron transfer rate fluctuates with CeO_2_-loading amount, and the yield of CO peaked on 30%CeO_2_ (selected from 10% to 70%). When exposed to UV light, CeO_2_ crystals could absorb more photons than visible light, whereas h-BN does not exhibit absorption in the UV range. The establishment of heterojunction expanded the effective wavelengths and improved the absorption capability in ultraviolet and visible light. The •OH species was detected by ESR analysis to identify the type of the heterostructure and the result of no newly generated •OH species was in accord with the II-scheme system.

#### 3.5.3. Summary

As a whole, the wider light harvesting range and longer separation time of charge pairs improved the photoreduction upshot. Overall, recent results on CO_2_ photoreduction by CeO_2_-based materials are presented in Table 5.

## 4. Other 3DOM Materials

**Figure 20:**
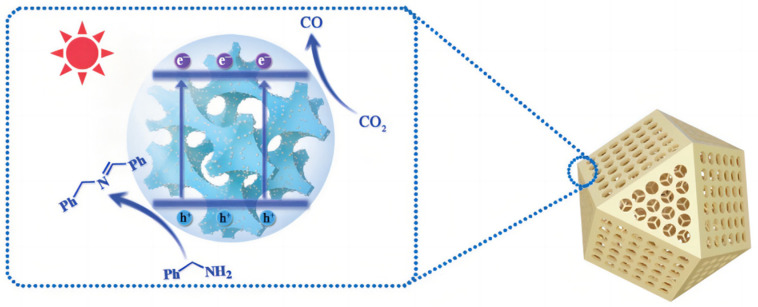
Proposed mechanism for the photocatalytic CO_2_ reduction on 3DOM CdS QD/NC. Schematic illustration of the photocatalytic CO_2_ reduction coupled with selective arylamine oxidation reaction system [143].

In order to introduce advanced porous structures to slow the self-aggregation of quantum dots, Wang et al. [143] devised 3DOM N-doped carbon (NC) to support CdS and ZnO QDs (Figure 20). They filled the interspace in an ordered PS microsphere template and then employed a pyrolytic treatment and in situ growth methods. Compared to bulky CdS, the 3DOM compounds have a larger cathodic current density and enhanced light harvesting, bearing a satisfactory carbon monoxide yield of 5210 μmol g^−1^ h^−1^. A three-dimensional SnO_2_ inverse opal structure was synthesized as gas sensors, soot oxidation catalyst and photoanode. The 3DOM BiVO_4_/SnO_2_ heterostructure was obtained by adding a BiVO_4_ precursor to fill the space between SnO_2_ skeleton and periodic PS template. The compatibility of energy states with SnO_2_ significantly reduced their photoluminescence intensity. Meanwhile, Au nanoparticles enhanced the slow photon effect, which in turn increased the incident light utilization efficiency.

Moreover, some photoreduction results on 3DOM materials are listed in Table 6. From the above analysis, we can infer the great potential of macroporous materials for further development in the field of photocatalysis.

## 5. Conclusions

The photoconversion of CO_2_ into solar fuels seems to curb greenhouse effect and resolve the energy crisis. In this review, the major research progresses of different metal oxide materials on solar-light-driven CO_2_ conversion to fuels were carefully summarized. TiO_2_, WO_3_, ZnO, Cu_2_O and CeO_2_ are the most common materials for photocatalysts among the numerous semiconductors. Even though considerable progress has been achieved, creating superb catalysts still presents several challenges. Researchers have presented treatment options to boost catalytic activity after apprehending the fundamental principles of objective reactions.

The main formulas for designing photocatalysts are as follows.

1. Absorb more photons to produce excitons.
2. Improve the migration efficiency of charge carriers.
3. Reduce the recombination rate of electron–hole pairs.
4. Uplift the absorption capacity of CO_2._

In terms of material selection, it is wise to choose metal oxides as one of the active components because of their relatively suitable *E*g and CB bands. From the perspective of morphology, compared with the solid and regular morphology, the structure of hollow ordered pores can promote the absorption of reactants and the desorption of products. A larger contact area between the catalyst and reactants and more active sites for the reaction can be provided. From the point of view of light absorption, the CB or VB positions can be adjusted after ion doping, which affects *E*g and influences the position of spectral absorption; the slow photon effect in the 3DOM structure can also improve the light utilization. As for charge separation efficiency, it can be promoted at the interface of the heterojunction, and photonic crystals can also improve their separation efficiency by shortening the distance of charge movement to the interface. Numerous attempts have been made in the synthesis of metal oxide photocatalysts, which can yield CO, CH_4_, HCHO, HCOOH, CH_3_OH and C_2_H_5_OH. However, it remains a great challenge for current photocatalysts to satisfy actual industrial production demands. The efficiency and selectivity for target products cannot meet the requirements for industrial and commercial implementation.

To sum up, the migration–separation efficiency of photoinduced pairs is important to improve the catalytic activity. Scholars should concentrate on the multiple advantages of photonic crystals to design catalysts with better performance based on them. This review provided a wealth of experience and ideas for the exploitation of photocatalyst material selection, morphology control and active site design. There is still a considerable work to conduct in converting CO_2_ from solar energy to fuel, and we believe more significant breakthroughs can be achieved regarding the efficiency, mechanism and durability of the photocatalyst.

## Figures and Tables

**Table 1:** Summary of the recent results of the photoreduction of CO_2_ using TiO_2_-based materials.

Type of Enhancement	Photocatalyst	Description	Conditions (Lamp, Cutoff Filter)	Product Yield/ μmol·g^−1^·h^−1^	Refs.
Heterojunction	Ag-NPs/TiO_2_	Core/shell	200 W Hg	CO 983 CH_4_ 9.73	[60]
Z-scheme	CuCo_2_S_4_@3B-TiO_2_	CCS yolk–shell hollow spheres, Bi-modified TiO_2_	300 W Xe	CO 25.5 CH_4_ 42.2	[56]
	Ag-Cu_2_O/TiO_2_	P25		CO 13.19 CH_4_ 1.74	[61]
S-scheme	Fe@TiO_2_/BCN	Fe@TiO_2_/Boron Carbon nitride	300 W Xe 400 nm	CH_4_ 24.7	[54]
p–n heterojunction	ZnFe_2_O_4_/TiO_2_	Spherical and irregular shapes	500 W Xe	CH_3_OH 75.34	[51]
Ion doping	Co-doped TiO_2_/rGO	Equiaxed grain morphologies	500 W Xe	CH_3_OH 133.7	[58]
3DOM	3DOM Pt@CdS/TiO_2_		420 nm irradiation light, UV cutoff filter	CH_4_ 36.8	[62]
	AuPd/3DOM-TiO_2_		300 W Xe	CH_4_ 18.5	[28]
	Pt_2_/3DOM-SrTiO_3_		300 W Xe	CH_4_ 26.7	[46]
	g-C_3_N_4_/Pt/3DOM TiO_2_@C		300 W Xe 420 nm	CH_4_ 65.6	[45]
	SnS_2_/3DOM SrTiO_3_		300 W Xe 420 nm	CH_4_ 12.5	[63]
	Cu_0.01_/3DOM TiO_2_		Xe (320–780 nm)	CH_4_ 43.5	[48]
	MoS_2_/3DOM TiO_2_		300 W Xe 420 nm	CH_4_ 11.6	[47]
	3DOM CeO_2_/TiO_2_		300 W Xe 420 nm	CO 3.73	[21]

**Table 2:** Summary of the recent results on the photoreduction of CO_2_ by WO_3_-based materials.

Type of Enhancement	Photocatalyst	Description	Conditions (Lamp, Cutoff Filter)	Product Yield/ μmol·g^−1^·h^−1^	Refs.
Heterojunction	Pd-Au/TiO_2_–WO_3_	0.5%Pd−0.1 wt%Au	400W Hg	CO 225.5 CH_4_ 15.1	[84]
	Bi_2_WO_6_/Si	Bi_2_WO_6_: Si = 1:1	300 W Xe 420 nm	C_2_H_5_OH 201	[85]
S-scheme	CdS/WO_3_	CdS nanoparticles at the WO_3_ surface	300 W Xe 420 nm	CO 35.625 CH_4_ 3.75	[86]
	WO_3_/CsPbBr_3_	3D porous melamine foam-supported	300 W Xe	CO+CH_4_ 600	[82]
Z-scheme	WO_3_-TiO_2_/Cu_2_ZnSnS_4_	Mesoporous ternary heterostructure	400 W Xe420 nm	CO 15.37 CH_4_ 1.69	[87]
	WO_3_/g-C_3_N_4_	3D/2D hollow microspheres	35 W HID car lamp and 300 W Xe (H_2_ reduction)	CO 145 CH_4_ 133	[88]
	TiO_2_/WO_3_/Pt	1D continuous fibrous structure	300W Xe	H_2_ 128.66	[89]
	CuO Dots/WO_3_	CuO quantum Dots/WO_3_ nanosheets	300 W Xe400 nm	CO 1.58	[81]
Ion doping	3%Mo-WO	Mo-doped WO_3_·0.33H_2_O nanorods	500 W Xe	CH_4_ 5.3	[83]
3DOM	g-C_3_N_4_/3DOM-WO_3_	300 W Xe 420 nm	visible light (≥420 nm)	CO 48.7 CH_4_ 7.5	[80]

**Table 3:** Summary of the recent results on the photoreduction of CO_2_ by WO_3_-based materials.

Type of Enhancement	Photocatalyst	Description	Conditions (Lamp, Cutoff Filter)	Product Yield/ μmol·g^−1^·h^−1^	Refs.
Heterojunction	Cu-ZnO Cel-T	Cellulose template designed porous ZnO	300 W Xe	CO 30.17	[102]
	OD-ZnO/C	Carbon dots, spherical morphology	400 W Xe	CO 118.8(600 °C)	[103]
	Pt/ZnO–ZnS	Porous ZnS–ZnO	300 W Xe, 420 nm	CH_3_OH 81.8	[90]
Z-scheme	Ag-Cu_2_O/ZnO	0.6Ag−0.4Cu_2_O ZnO nanorods	300 W Xe	CO 3.36	[104]
	g-C_3_N_4_/ZnO/graphene aerogel	Porous ZnO nanosphere	300 W Xe, 420 nm	CO 33.87	[93]
	ZnO/ZnS/g-C_3_N_4_	Spherical ZnS, ZnO nanoflowers	300 W	H_2_ 301	[94]
p–n heterojunction	ZnO/CuO/Zeolite	ZnO/CuO in NaAlSiO_4_ channels	two 20 W halogen lamps	H_2_ 62.3 HCOOH 907	[18]
Ternary catalyst	R-ZnO@ LDH	Core–shell Structure, belt-like ZnO hierarchical LDH	300 W Xe	CH_4_ 11.4	[105]
3DOM	CoO_x_/N-C-ZnO	N, C doped	420 nm	CO 26.4	[99]

**Table 4:** Summary of the recent results on the photoreduction of CO_2_ by Cu_2_O-based materials.

Type of Enhancement	Photocatalyst	Description	Conditions (Lamp, Cutoff Filter)	Product Yield/ μmol·g^−1^·h^−1^	Refs.
Heterojunction S-scheme	Cu_2_O/CNPs	Coal-based carbon nanoparticles, multilayer graphene lattice structure	300 W Xe	CH_3_OH 24.86	[120]
	g-C_3_N_4_ foam/Cu_2_O QDs	3D g-C_3_N_4_ foam, 0D Cu_2_O QDs	300 W high-pressure Hg	CO 8.182	[123]
	Cu_2_O/rGO	Rhombic dodecahedra	300 W Xe 420 nm	CH_3_OH 17.77	[117]
	Ag/Cu_2_O@rGO	Octahedral Cu_2_O	300 W Xe 380 nm	CH_4_ 82.6	[112]
Z-scheme	UiO-66-NH_2_/Cu_2_O/Cu	Octahedron UiO-66-NH_2_	300 W Xe	CO 4.54	[122]
p–n heterojunction	Mg(OH)_2_/CuO/Cu_2_O	Microwave-hydrothermal method	Liquid phase: two halogen lamps of 20W	CH_3_OH 6 HCHO 9	[118]
	Cu_2_O/SnS_2_/SnO_2_	Hierarchical flower-like SnS_2_/SnO_2_	300 W Xe	CO 3.18 CH_4_ 2.27	[119]
3DOM	3D porous Cu_2_O		300 W Xe, 420nm	CO 13.4 nmol cm^−2^h^−1^	[114]

**Table 5:** Summary of the recent results on the photoreduction of CO_2_ by CeO_2_-based materials.

Type of Enhancement	Photocatalyst	Description	Conditions (Lamp, Cutoff Filter)	Product Yield/ μmol·g^−1^·h^−1^	Refs.
Heterojunction	modified TiO_2_/rGO/CeO_2_	Sono-assisted, 0.75 wt% rGO and 1 wt% CeO_2_	UV light (a 15 W UV-C mercury)	CH_3_OH 106.83 C_2_H_5_OH 45.17	[53]
	S-C/In_2_O_3_-CeO_2_	S-doped, hollow hexagonal prisms with carbon coating	300 W Xe	CH_4_ 60.6	[136]
	CeO_2_/Fe_3_O_4_	G-C_3_N_4_ QDs (CN QDs) CeO_2_/Fe_3_O_4_ micro-Flowers (MFs)	UV-Vis light	CO 28.0 CH_4_ 9.5	[137]
	Ni/CeO_2_	Pure cubic fluorite structure CeO_2_	Xe 4.7 kW m-2 >500 °C	H_2_ 6.53 CO 6.27	[138]
	CeO_2_@Ti_3_C_2_TX	Layered Ti_3_C_2_TX nanosheets, high-density CeO_2_	300 W Xe	CH_3_OH 76.2 C_2_H_5_OH 33.7	[139]
Z-scheme	CoAl-LDH/CeO_2_	CeO_2_ and RGO on the flower-like CoAl-LDHs	ultraviolet (UV) light (200 W)	CO 5.5	[140]
p-n heterojunction	NiO/CeO_2_/rGO	CeO_2_ nanorods, hexagon-shaped NiO plates	300 W Xe	HCHO 421.09	[132]
Ternary composites	N-TiO_2_/CeO_2_/CuO	N-doped TiO_2_	2 Xe lamps (20 W each)	HCOOH 33	[141]
Ion doping	Fe-Ni@CeO_2_	Spherical Fe and hexagonal Ni-doped CeO_2_ nanorods	20 W white LED	CH_3_OH 293.29	[142]

**Table 6:** Summary of the recent results on the photoreduction of CO_2_ and H_2_O by 3DOM materials.

Type of Enhancement	Photocatalyst	Conditions (Lamp, Cutoff Filter)	Product Yield/ μmol·g^−1^·h^−1^	Refs.
Heterojunction	3DOM Au-CsPbBr_3_	300 W Xe 420 nm	CO 12.6 CH_4_ 2.1	[82]
	3DOM CdS QDs/N-doped carbon	visible light irradiation, acetonitrile solution	CO 5210	[143]
Water splitting to produce H_2_	CdS/Au/3DOM-SrTiO_3_	300 W Xe 420 nm	5.39 × 10^3^	[144]
	3DOM Pt/ZnS@ZnO	300 W Xe	87.6	[145]
	TiO_2_-Au-CdS	34 mW/cm^2^ UV light, 158 mW/cm^2^ visible light	1.81 × 10^3^	[146]

## Data Availability

Not applicable.
